# Dental discrepancies in black adolescents: evaluating impacts on well-being

**DOI:** 10.1590/1807-3107bor-2025.vol39.015

**Published:** 2025-02-07

**Authors:** Jean Érick LANGOSKI, Ana Claudia Lima de Oliveira MEIRA, Milton SANTAMARIA-JUNIOR, Carolina Carmo de MENEZES, Marcelo de Castro MENEGHIM, Silvia Amélia Scudeler VEDOVELLO

**Affiliations:** (a) Centro Universitário da Fundação Hermínio Ometto, Araras Dental School, Department of Orthodontics, Araras, SP, Brazil.; (b) Universidade Estadual Paulista – Unesp, School of Dentistry of Araraquara, Department of Orthodontics, Araraquara, SP, Brazil.; (c) Universidade Estadual de Campinas – Unicamp, Piracicaba Dental School, Department of Health Sciences and Child Dentistry, Piracicaba, SP, Brazil.; (d) Universidade Estadual de Campinas – Unicamp, Piracicaba Dental School,Department of Orthdontics, Piracicaba, SP, Brazil.

**Keywords:** Malocclusion, Adolescent, Esthetics, Dental, Orthodontics

## Abstract

The aim of this study was to compare the esthetic, functional, and psychosocial impact of mandibular crowding and maxillary midline diastema in black adolescents. A cross-sectional study was conducted with 420 black (brown and black, distinguished according to Brazilian Institute of Geography and Statistics – IBGE) adolescents aged 12 with normal occlusal relationships. Esthetic (OASIS) and functional/psychosocial (OHIP-14) impact related to the need for orthodontic treatment in groups with mandibular crowding and maxillary midline diastema, and those without these conditions: G1, without crowding and diastema (n 113); G2, without crowding and with diastema (n 67); G3, with crowding and without diastema (n 202); and G4, diastema, and crowding (n 38) were evaluated. Generalized linear models were estimated for the effects of diastema, crowding, and the interaction between them, with a significance level of 5%. There was no significant influence of crowding and diastema on the OHIP-14 (p>0.05). However, the groups with diastema had higher OASIS scores, irrespective of crowding (p<0.05). The maxillary midline diastema influenced the esthetic perception of black adolescents.

## Introduction

The need for orthodontic treatment is associated with functional and esthetic changes, and psychosocial consequences for the population in different stages of development.^1-5^ In this sense, the evaluation of malocclusion must be carried out in a multidimensional context, considering all possible determinant factors of malocclusion.^6^ In addition to clinical records, it is clear that the individual’s perception is an essential factor in oral health surveillance and is one of the guiding factors for planning and orthodontic treatment.^7-8^ The literature has previously suggested the negative influence of malocclusion on daily life.^9-11^ Individuals with severe malocclusion feel less satisfied with the appearance of their mouth and teeth. Consequently, they feel shy when they smile.^2,12^

Excess space, or conversely, the presence of spaces in the dental arch, is considered malocclusion in permanent dentition.^13,14^ The tooth-bone discrepancy, therefore, includes incompatibility between the bone morphology and tooth mass. Thus, diastema and crowding are risk factors for negative oral health-related quality of life (OHRQoL)^15^, especially because of their potential to influence both esthetics and function. Malocclusion in the smile zone has a negative impact on the social and emotional life of individuals, irrespective of the population and stages of occlusal development.^15,16^

Some etiological factors contribute to the development of maxillary midline diastema, including tooth size and tooth-bone discrepancies, mesiodens, and lip brake.^17^ It is interesting to highlight the perception of diastema, which can be seen as a need for treatment or an esthetic trait, and may be influenced by culture, age group, and racial origin.^18^ Om contrast, although mandibular crowding is a recurrent subject in the literature, its etiology and relapses have led to controversies since its prevalence represents one of the most frequent complaints of patients who seek an orthodontist.^19^

It is evident that while the impact of various aspects related to malocclusion are well-documented in the white population, the topic is still relatively unknown in the black population. Studies have suggested that adolescents with brown or black skin are more likely to experience severe malocclusion compared with their white counterparts.^1,9^ Social, economic, and demographic factors may influence the severity of malocclusion and, consequently, negatively affect quality of life.^9^ Malocclusion result from complex interactions of various factors, significantly impacting an individual’s environmental aspects. Therefore, black populations consistently experience poorer oral health outcomes compared with other racial groups.

Moreover, the socioeconomic context can influence the severity of malocclusion, potentially aggravating this population’s perception of oral health.

Ethnicity is a predisposing factor in determining the size of the dental arches and, consequently, in the occlusal characteristics of individuals,^20,21^ including the prevalence of anterior occlusal alterations.^22,23^ Considering the hypothesis that dental discrepancies significantly affect adolescents’ well-being, the present study compared the esthetic and functional impact of mandibular crowding and maxillary midline diastema in black adolescents.

## Methodology

### Participants

This cross-sectional study was approved by the Research Ethics Committee (58303616.0.0000.5385). The study included only black adolescents (brown and black), distinguished according to Brazilian Institute of Geography and Statistics – IBGE^24^ in Class D according to Brazilian Economic Classification Criterion (CCEB), of both sexes, aged 12, from public schools in Salvador (Bahia, Brazil). Previous studies considering this same database have previously been published.

The sample size was calculated to provide a test power of 80% for the main effects of crowding (yes or no) and diastema (yes or no) and for the interaction between them (crowding x diastema), with a significance level of 5% and small effect size. The sample size was calculated using the GPower program (Franz Faul, Christian-Albrechts-Universitat, Kiel, Germany) 26 that indicated a minimum sample of 420 adolescents.

The probabilistic sampling was performed by conglomerates. The distribution of 12-year-old adolescents in each administrative district of Salvador was determined from information provided by the Municipal Secretary of Education. The sample was stratified according to the administrative district, and in the first phase, schools were selected using a simple randomization procedure. Likewise, adolescents were selected for the sample using a simple randomization procedure in the second phase.

### Eligibility criteria

Exclusively, adolescents who provided the Term of Informed Consent Form signed by their parents or guardians and the Minor’s Assent Form were included in the study. The following clinical eligibility criteria were considered^27^:

- Permanent dentition;
- Class I molar relationship;
- Normal horizontal and vertical overbite (ranging from 0 to 3mm); anf
- Normal transverse relationship (indicated by the absence of crossbite).

Adolescents who did not meet the eligibility criteria or reported current or previous orthodontic treatment, systemic diseases, communication problems, or neuromotor disorders were excluded.

### Clinical evaluation

The study was designed to evaluate the esthetic, functional, and psychosocial impact related to the need for orthodontic treatment in groups with tooth size-arch length discrepancy (mandibular crowding and maxillary midline diastema) and those without tooth size discrepancy.

The adolescents were clinically evaluated inside the schools under natural light by a single calibrated evaluator. Clinical evaluation of mandibular crowding in the anterior segment was observed only in the mandibular arch, including the four permanent incisors. Crowding was considered when the space between the right and left canines was insufficient to accommodate the four incisors in alignment.^28^ Midline maxillary diastema was clinically recorded in millimeters from the height of the line of adjacent teeth or at the point of greatest convexity on the proximal surface. Values ≤ 1 mm were considered as the absence of diastema and > 1 mm as the presence of diastema.^28^

**Figure 1:**
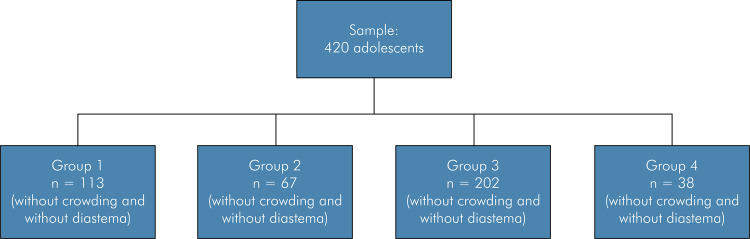
Flowchart of study design.

Figure 1 shows the flowchart of the study design. The sample was stratified into Group 1 (without mandibular crowding and without maxillary midline diastema, n = 113), Group 2 (without mandibular crowding and with maxillary midline diastema, n = 67), Group 3 (with mandibular crowding and without maxillary midline diastema, n = 202), and Group 4 (with mandibular crowding and maxillary midline diastema, n = 38).

### Esthetic and functional evaluation

The Orthodontic Aesthetic Subjective Impact Score (OASIS)^29^ was used to determine the esthetic impact of mandibular crowding and maxillary midline diastema.^5,14^ The OASIS contains five items in the first part, with three answer options, and the responses are quantified and scored in ascending order according to the Likert scale. The second part includes the Aesthetic Component of the Orthodontic Treatment Needs Index (IOTN-AC).^30^ The AC-IOTN was used to assess psychosocial needs by means of an attractiveness scale illustrated with ten color photographs arranged in a descending and continuous level; image one represents the most attractive oral condition, and image ten is the least attractive. After guidance from the examiner, the interviewees completed their self-assessment, identifying the level of esthetic impairment according to the scale images, which they considered like their respective smiles. The result of the OASIS was obtained by adding the instrument responses to the value of the image selected in the IOTN-AC to produce a single score, and the total score of the instrument was used in the analysis.

The Oral Health Impact Profile (OHIP-14)^31^ was used to assess the influence of occlusal changes on adolescents’ daily activities, indicating the functional and psychosocial impact of the problem. The OHIP-14 contains 14 questions that assess the following aspects: a) functional limitation; b) physical pain; c) psychological discomfort; d) physical incapacity; e) psychological incapacity; f) social incapacity, and g) social disadvantage. The answers are recorded according to the scale of codes and their respective meanings: 0 = “never”; 1 = “rarely”; 2 = “sometimes”; 3 = “fairly often”; 4 = “always”. Likewise, the total score was used for statistical analysis.

### Calibration of operators

An examiner trained in orthodontics performed the examinations in schools under artificial light. Before starting the experimental phase, a calibration was performed to obtain acceptable consistency for all clinical conditions. An experimental phase was followed by a theoretical discussion. During training and calibration, the inter- and intra-examiner agreement was estimated by the intraclass correlation coefficient (ICC) for mandibular crowding and maxillary midline diastema, with an acceptable cutoff value (Kappa = 0.92 and 0.95, respectively).

### Statistical analysis

Initially, descriptive and exploratory data analyses of were carried out. Absolute and relative frequencies, means, standard deviations, medians, and minimum and maximum values were used. Then, generalized linear models were estimated for the effects of diastema, mandibular crowding, and the interaction between them. Sex was considered a covariate in the models. Analyses were performed using the R program (R Foundation for Statistical Computing, Vienna, Austria), with a significance level of 5%.

## Results

The study included 420 black adolescents. Table 1 presents the descriptive analysis of the studied variables.

**Table 1:** Sample characteristics (n = 420).

Variable	Groups			
	Group 1*	Group 2**	Group 3***	Group 4****
n (%)	113 (26.9%)	67 (16.0%)	202 (48.1%)	38 (9.0%)
Gender n(%)				
Male	42 (37.2%)	26 (38.8%)	90 (44.6%)	19 (50.0%)
Female	71 (62.8%)	41 (61.2%)	112 (55.4%)	19 (50.0%)

*without crowding and without diastema; ** without crowding and with diastema; *** with crowding and without diastema; **** with crowding and diastema.

**Figure 2:**
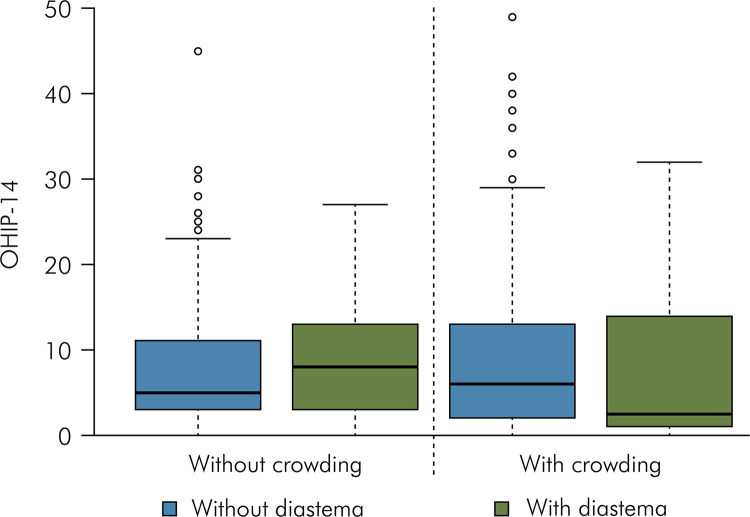
Box plot of esthetic impact (OHIP-14 scores) considering the presence of mandibular crowding and midline diastema (n = 420).

Table 2 and Figure 2 present the OHIP-14 scores according to the presence of mandibular crowding and diastema. There was no significant effect of crowding and diastema in the OHIP-14.

**Table 2:** Functional and psychosocial impact (OHIP-14) and presence of mandibular crowding and midline diastema (n = 420).

Mandibular crowding	Midline diastema			
	Without		With	
	Mean (standard deviation)	Median (minimum and maximum value)	Mean (standard deviation)	Median (minimum and maximum value)
Without	8.5 (8.5) Aa	5.0 (0.0-45.0)	9.1 (7.1) Aa	8.0 (0.0-27.0)
With	9.0 (9.2) Aa	6.0 (0.0-49.0)	8.0 (9.8) Aa	2.5 (0.0-32.0)

p(diastema) = 0.8894; p(crowding) = 0.8560; p(interaction) = 0.5546; Same letters (Capital letters horizontally and lowercase vertically) indicate that there are no statistically significant differences (p > 0.05).

**Figure 3:**
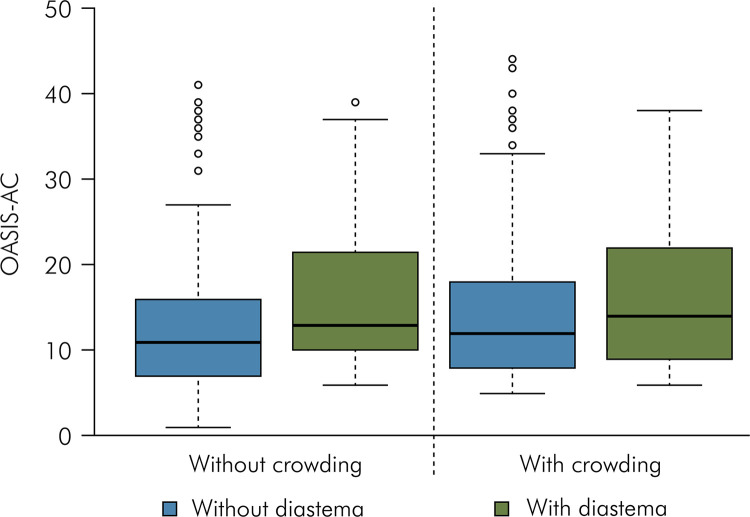
Box plot of Esthetic impact (OASIS scores) considering the presence of mandibular crowding and midline diastema (n = 420).

Table 3 presents the OASIS scores according to the presence of mandibular crowding and diastema. The OASIS scores were significantly higher when the adolescents presented midline diastema, irrespective s of mandibular crowding (p < 0.05) (Figure 3). Group 4 (with mandibular crowding and diastema) had higher OASIS scores than Group 1 (without mandibular crowding and without diastema) and Group 3 (with mandibular crowding and without diastema) (p < 0.05). Group 2 (without mandibular crowding and with diastema) had higher scores than those without the two conditions (p < 0.05).

**Table 3:** Esthetic impact (OASIS) and presence of presence of mandibular crowding and midline diastema (n =4 20).

Mandibular crowding	Midline diastema			
	Without		With	
	Mean (standard deviation)	Median (minimum and maximum value)	Mean (standard deviation)	Median (minimum and maximum value)
Without	13.9 (8.9) Ba	11.0 (1.0-41.0)	16.3 (8.7) Aa	13.0 (6.0-39.0)
With	14.4 (8.2) Ba	12.0 (5.0-44.0)	17.4 (9.6) Aa	14.0 (6.0-38.0)

p(diastema) = 0.0017; p(crowding) = 0.3359; p(interaction) = 0.8631; Distinct letters (Capital letters horizontally and lowercase vertically) indicate statistically significant differences (p ≤ 0.05).

## Discussion

The present study evaluated the aesthetic, functional, and psychosocial impact of mandibular crowding and maxillary midline diastema in black adolescents. Our study revealed that irrespective of mandibular crowding, midline diastema had a negative effect on the esthetic perception of adolescents. Including a sample of black adolescents (specifically, individuals black and brown, according to IBGE24) contributes to comprehending the aesthetic, functional, and psychosocial outcomes of malocclusion in the smile region within this particular demographic.

This study was part of an epidemiological survey conducted with black adolescents.^9,25^ The adolescents, with the same socioeconomic conditions,^24^ came from Bahia (Brazil), a region with the highest level of African ancestry (50.8%) in the world, considered the blackest city outside the African continent.^25,32^ Significantly, 80.8% of Bahia’s population (composed of black and brown individuals) declares itself as being black ^24^This proportion is the highest among all Brazilian states, surpassing the national average of 55.9% and that of the Northeast region, 73.9%. These insights were derived from the Continuous National Household Sample Survey (PNADC) by the Brazilian Institute of Geography and Statistics (IBGE) in 2022.^24^

It is important to highlight that in our study, only adolescents with normal occlusal relationships were included to avoid bias in evaluating the impact of the mandibular crowding and maxillary midline diastema. To establish the relationship and the attributes of the instruments used to justify the methodological choice, the OASIS is used to evaluated the degree of dissatisfaction of the interviewee about the adolescent’s teeth, considering smile esthetics. Based on the occlusal condition, the OHIP-14 was used to address the functional and psychosocial impact on the individual.^33^

The isolated presence of mandibular crowding did not demonstrate any association with adolescents’ perception, reaffirming that anterior crowding, in isolation, has a minimal effect on OHRQoL.^34^ However, the effects can be significant when evaluating the overall severity of malocclusion, using scores encompassing a possible pattern of midline diastema and/or mandibular crowding.^35,36^In the present study, the fact that anterior malocclusion was evaluated separately (positive or negative discrepancy) may justify the result since these conditions may have no or minimal impact on functional abilities.^34^ Whereas as it was impossible to determine the severity of the mandibular crowding. This is a likely hypothesis for why the OHRQoL was not affected. Future studies should include only cases with moderate or severe crowding to investigate the impact of crowding severity.

In the same way, maxillary midline diastema impacted the esthetic perception of adolescents. The findings corroborated those of previous studies that showed that diastemas equal to or greater than 1mm negatively dentofacial esthetics,^37^ and the larger and more mesially located the diastema, the less attractive the smile would be considered.^38^It is important to highlight that the maxillary midline diastema is a clinical sign with multifactorial etiology.^39,40^ The perception of midline diastema, as a need for orthodontic treatment or as an esthetic trait, may vary with culture, age range, and racial origin.^18,40^ Therefore, the findings indicate that maxillary diastema may have implications for personal and social issues, potentially resulting in constraints and setbacks in the social integration of individuals affected.^41^

The present study was conducted with black adolescents of the same socioeconomic status who resided in the urban area of the city of Salvador (Bahia, Brazil). It is necessary to note that socioeconomic, cultural, and environmental conditions play a pivotal role in influencing the social determinants of health and can significantly impact the health status of a population. These factors, in conjunction with genetic and racial factors, are particularly prevalent in the black population, characterizing the environment and potentially contributing to the severity of malocclusion and dental discrepancies in adolescents.^1,9^

Conducting additional epidemiological investigations involving black populations should be considered a priority. In this sense, our findings strengthen the need for further studies to evaluate adolescents’ perceptions of oral health. This is particularly important considering the distinct impact of each component of malocclusion on individuals’ esthetic satisfaction and daily activities, and understanding the particular esthetic perception exhibited by each racial group plays an increasingly important role in the results of orthodontic treatments. This indicated that the perception of occlusal alteration could influence access to the local healthcare system.

## Conclusion

Maxillary midline diastema influences the esthetic perception of black adolescents. Mandibular crowding and maxillary midline diastema do not affect the functional and psychosocial aspects of adolescents’ daily life, highlighting the role played by tooth discrepancy in esthetics.
